# PsAPASH: a rare and recent autoinflammatory syndrome associated with hidradenitis suppurativa^☆^^☆☆^

**DOI:** 10.1016/j.abd.2019.02.012

**Published:** 2020-01-21

**Authors:** Raissa de Lima Gadelha, Renata da Silveira Rodrigues Paiva, Esther Bastos Palitot, Joanne Elizabeth Ferraz da Costa

**Affiliations:** Department of Dermatology, Hospital Universitário Lauro Wanderley, João Pessoa, PB, Brazil

**Keywords:** Acne vulgaris, Arthritis, psoriatic, Hidradenitis suppurativa, Pyoderma gangrenosum

## Abstract

Hidradenitis suppurativa is a chronic inflammatory skin disease, which affects 1% of the population, being more common in young, obese and smokers, and mainly affects armpits and groin, with formation of pustules, nodules, abscesses, scars and fistulas. Recently, its association with other autoimmune diseases such as psoriasis, psoriatic arthritis, pyoderma gangrenosum, pyogenic arthritis and ulcerative colitis have been reported. These associated forms are usually resistant to standard treatment, with immunobiologicals as promising therapy. The case of a rare form of association is reported, with only one case previously described in the literature: psoriasis arthritis, pyoderma gangrenosum, acne and hidradenitis suppurativa.

## Introduction

Suppurative hidradenitis is a chronic inflammatory skin disease with pathophysiology based on follicular occlusion of the pilosebaceous unit and innate deregulated immune response.^1^, ^2^

Suppurative hidradenitis and acne may be essential components of the auto-inflammatory syndromes which are described: Pyoderma gangrenosum, Acne and Pyogenic Arthritis (PAPA), pyoderma gangrenosum, acne and hidradenitis suppurativa (PASH), Pyoderma gangrenosum, Acne, Pyogenic arthritis and Hidradenitis Suppurativa (PAPASH) and Pyoderma gangrenosum, Acne and Spondyloarthritis (PASS). Recently it was added to the spectrum the syndrome composed of Psoriatic Arthritis, Pyoderma gangrenosum, Acne and Hidradenitis Suppurativa (PsAPASH) and Pyoderma gangrenosum, Acne and ulcerative Colitis (PAC).^3^

Auto inflammatory syndromes are defined by an aberrant innate immune system and absence of circulating autoantibodies and autoreactive T-cells and it is believed that recurrent episodes of neutrophilic inflammation are mediated by interleukin 1.^4^ The symptoms are usually severe and may not respond to standard treatments and thus have devastating physical and psychological consequences for the affected patients.^5^

## Case report

A 22 year-old female patient reported painful lesions in the armpits 3 years later with dissemination to the groin and breasts. One year ago, acne lesions appeared in addition to lower limb ulcers and scaly lesions on the scalp appeared for 3 months and were referred to our HULW Dermatology service. He also reported joint pains in hands, wrists, knees and lumbar spine with morning stiffness of 30 min.

**Figure 1:**
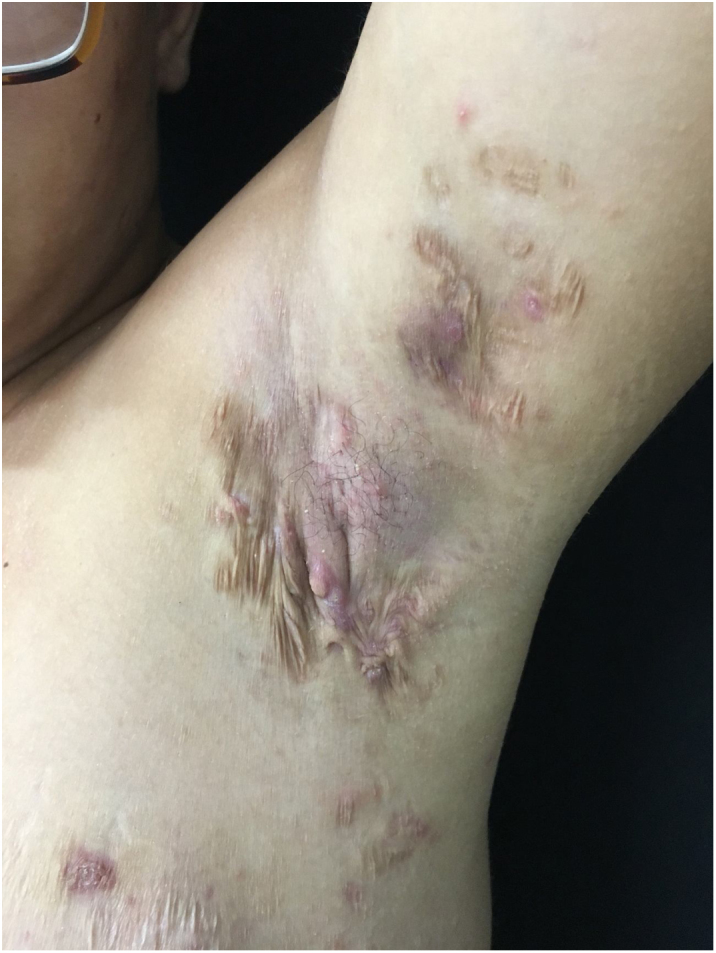
Injuries suggestive of hidradenitis suppurativa in the axilla: nodules, scars and fibrosis.

**Figure 2:**
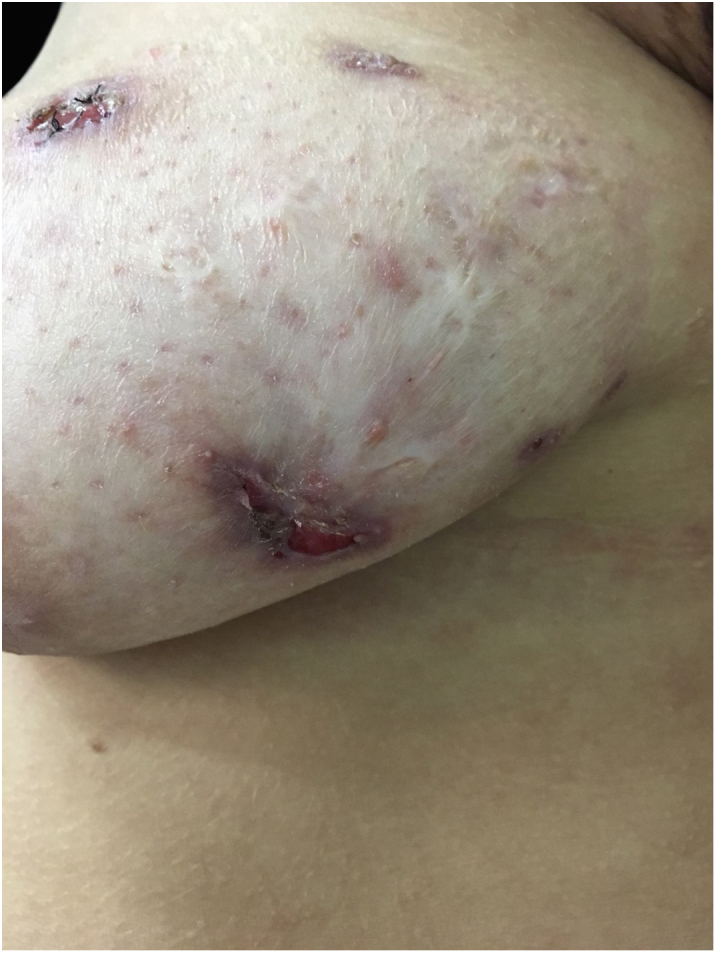
Lesions suggestive of pyoderma gangrenosum in the left breast: ulcers with violaceous and undermined borders.

**Figure 3:**
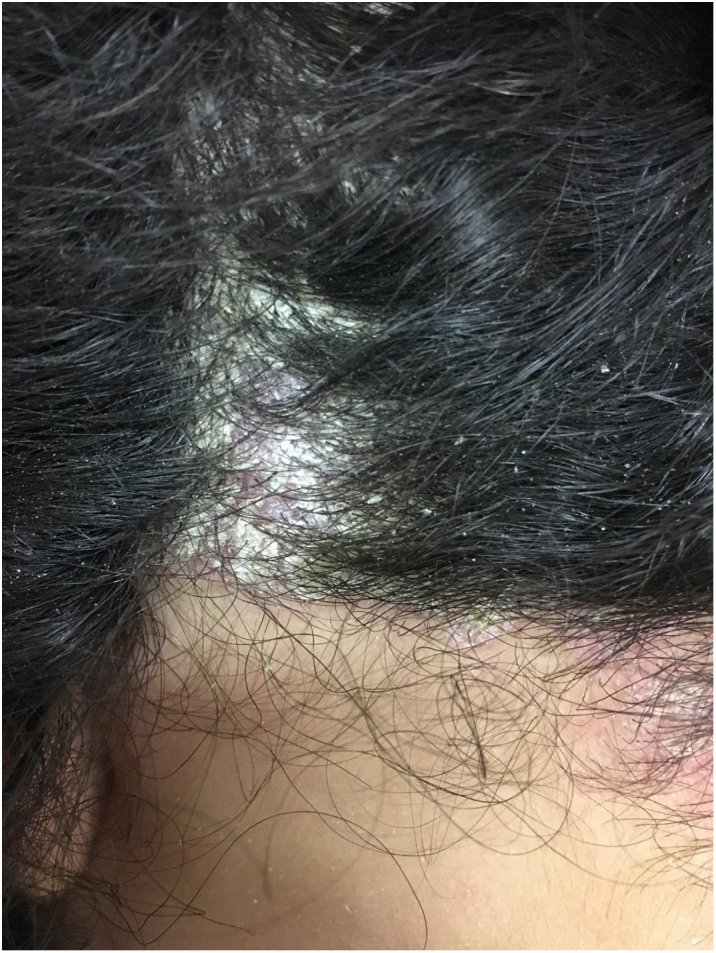
Erythematous scaly lesions on the scalp suggestive of psoriasis.

**Figure 4:**
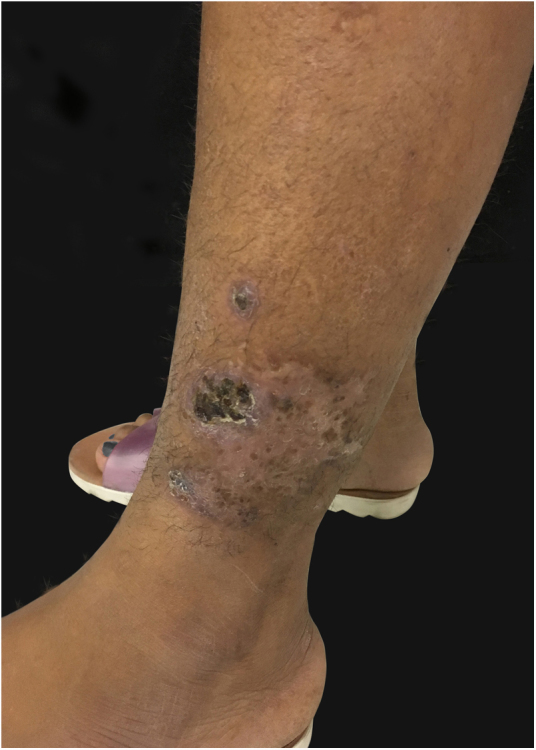
Ulcerated cicatricial lesion in lower left limb of pyoderma gangrenosum.

At the examination he had Grade 2 acne on the face; abscesses, fistulae and fibrosis in armpits and groin compatible with Hurley's Grade III suppurative hidradenitis; scaly erythematous scaly plaques suggestive of psoriasis and pseudotinha amiantácea; and purulent painful nodules and ulcers with violaceous, protruding and undermined left breast tendrils suggestive of pyoderma gangrenosum (Figure 1, Figure 2, Figure 3, Figure 4).

The patient was medicated with capillary solution of salicylic acid and betamethasone dipropionate and antisecretory shampoo, in addition to clindamycin 300 mg every 8 h and metformin 500 mg twice daily.

The anatomopathological fragment of the scalp revealed epidermis with psoriasiform acanthosis, paraaceratosis, hypogranulosis and intracranial microabscesses, a dermis with inflammatory infiltrate of the perivascular lymphohistiocytic, compatible with psoriasis. Already the fragment of the left breast showed cutaneous ulcer with dense and diffuse mixed inflammatory infiltrate, with abscessed foci, focal areas of necrosis and frequent leukocytoclasia.

He presented laboratory tests, such as non-reactive FAN, VHS 54, Negative Rheumatoid Factor, serologic tests for Hepatitis B and C negative, PPD 0 mm, Chest X-Ray without changes. Based on the CASPAR criteria, it presents a diagnosis of psoriatic arthritis (peripheral arthritis, current psoriasis and negative rheumatoid factor), and was referred to the Rheumatology department for interdisciplinary follow-up.

**Figure 5:**
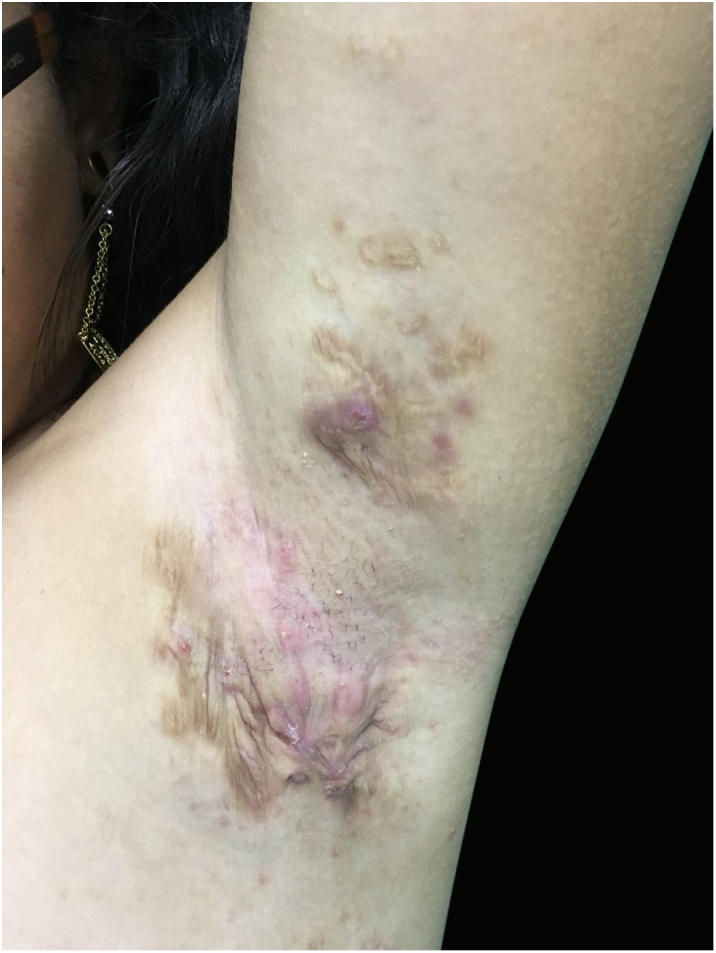
Lesions in the left axilla presenting improvement of the picture after 2 months of use of Adalimumabe SC.

**Figure 6:**
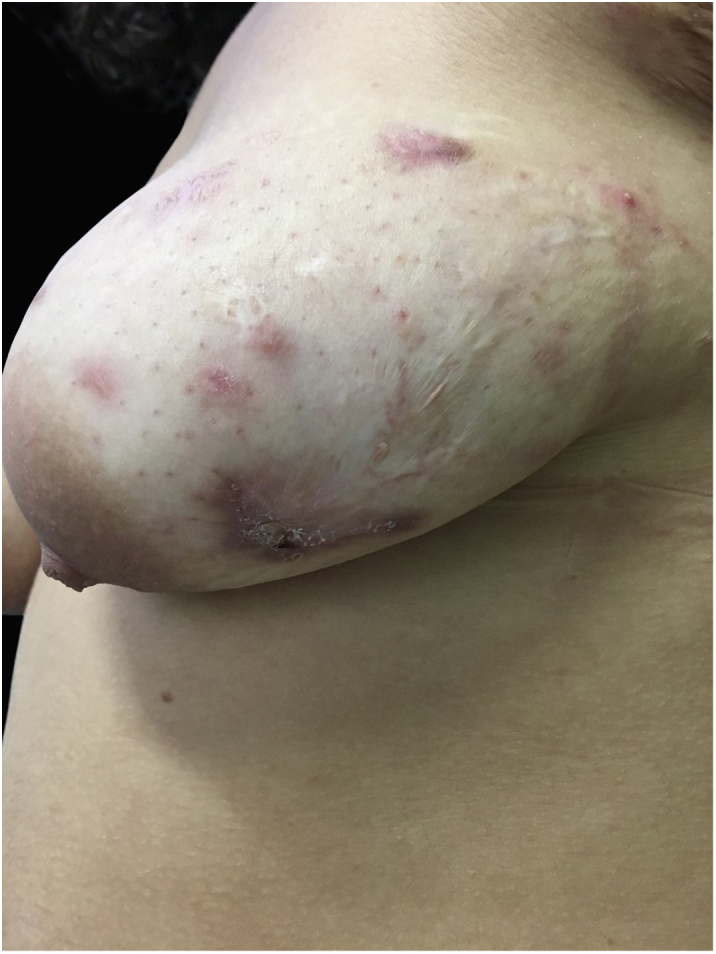
Lesions in the left breast presenting improvement of the picture after 2 months of use of Adalimumabe SC.

The patient did not present improvement with initial treatment and then started using Adalimumab in a scheme for hidradenitis suppurativa and presented an important improvement of the picture at the consultation after 2 months (Figure 5, Figure 6), evidenced by the decrease in Sartorius score (underarm, previous: 55, after 2 months: 49).

The schedule was performed with 4 syringes, subcutaneously, of Adalimumab (40 mg) at week 0 and 2 syringes at week 2 (induction phase), followed by 1 syringe every 7 days (maintenance phase). The same goes on regular quarterly monitoring in our service.

## Discussion

Suppurative hidradenitis is a chronic inflammatory disease of the skin, clinically defined by nodules and recurrent painful abscesses that lead to the formation of fistulas, nodules and scars. It occurs in 1% of the population, is more frequent in women and tends to resolve at menopause.^6^

Locally recurring lesions may be excised or treated with a laser. Disseminated lesions, however, can be treated with radical surgery, topical or systemic antibiotics (tetracycline or clindamycin with rifampicin), oral retinoids, dapsone and biological agents (adalimumab and infliximab). Pain management, weight loss, tobacco abstinence and treatment of superinfections are also important recommendations for all patients.^7^

Suppurative hidradenitis associated with autoinflammatory syndromes is often severe (Hurley II, III) and does not respond to many of the usual treatments.^8^

Because of the likely common pathogenesis involving IL-1-induced inflammation, collective experiments to date indicate that IL-1 and TNF-targeted therapies represent the most successful treatment solution for prolonged remission. TNF-blocking agents, adalimumab and infliximab, have achieved good control of suppurative hidradenitis lesions and, thus, reinforce the role of a deregulated innate immune response in the disease pathogenesis.^3^

The autoinflammatory syndrome composed of the association of psoriatic arthritis, pyoderma gangrenosum, acne and hidradenitis suppurativa (PsAPASH) was described in 2015 and there was no other similar description in the literature.^7^ As in the case described, our patient was treated with Adalimumab and presented in remission of the disease.

No genetic mutation associated with PsAPASH has yet been identified, but the other syndromes are associated with the genetic mutation in the coding region of PSTPIP1 as well as with increased repetitions of CCTG in the promoter region of PSTPIP1, leading to its deregulation and predisposition to cutaneous neutrophilic inflammation.^9^

Thus, it is very important that dermatologists know about autoinflammatory syndromic hidradenitis, including typical lesions of pyoderma gangrenosum, hidradenitis suppurativa, psoriasis and acne, and laboratory findings suggestive of systemic inflammation to establish clinical prevalence and early treatment.^7^

## Financial support

None declared.

## Authors’ contributions

Raissa de Lima Gadelha: Statistical analysis; conception and planning of the study; elaboration and writing of the manuscript; obtaining, analysis, and interpretation of the data; intellectual participation in the propaedeutic and/or therapeutic conduct of the studied cases; critical review of the literature; critical review of the manuscript.

Renata da Silveira Rodrigues Paiva: Approval of the final version of the manuscript; effective participation in research orientation; intellectual participation in the propaedeutic and/or therapeutic conduct of the studied cases.

Esther Bastos Palitot: Approval of the final version of the manuscript; effective participation in research orientation; intellectual participation in the propaedeutic and/or therapeutic conduct of the studied cases.

Joanne Elizabeth Ferraz da Costa: Approval of the final version of the manuscript; effective participation in research orientation; intellectual participation in the propaedeutic and/or therapeutic conduct of the studied cases.

## Conflicts of interest

None declared.
